# Burns to be alive: a complication of transcutaneous cardiac stimulation

**DOI:** 10.1186/s13054-014-0622-x

**Published:** 2014-11-12

**Authors:** Xavier Muschart

**Affiliations:** CHU Dinant Godinne, UCL-NAMUR 1, av. Gaston Therasse, 5530 Yvoir, Belgium

External cardiac pacing devices (ECPDs) are commonly employed in emergency situations. The main indication for their use is untolerated bradycardia, especially in cases where other medical treatments have no effect [1]. Although the efficacy and safety of ECPDs are well documented, a classic side effect associated with their use is pain secondary to the electrically induced muscular contraction. Therefore, correct sedation-analgesia is critical for avoiding pain when reaching the correct voltages required for effective electrostimulation.

Here, we report the case of an 86-year-old patient with third-degree skin burns secondary to the use of an ECPD (a Zoll M Series Biphasic® defibrillator along with Stat Padz Multi-Function® adult electrodes with high viscosity polymer gel; Zoll Medical Corporation, USA) (Figure 1). To date, this observation represents the most serious ECPD-associated adult complication described in the literature.

**Figure 1:**
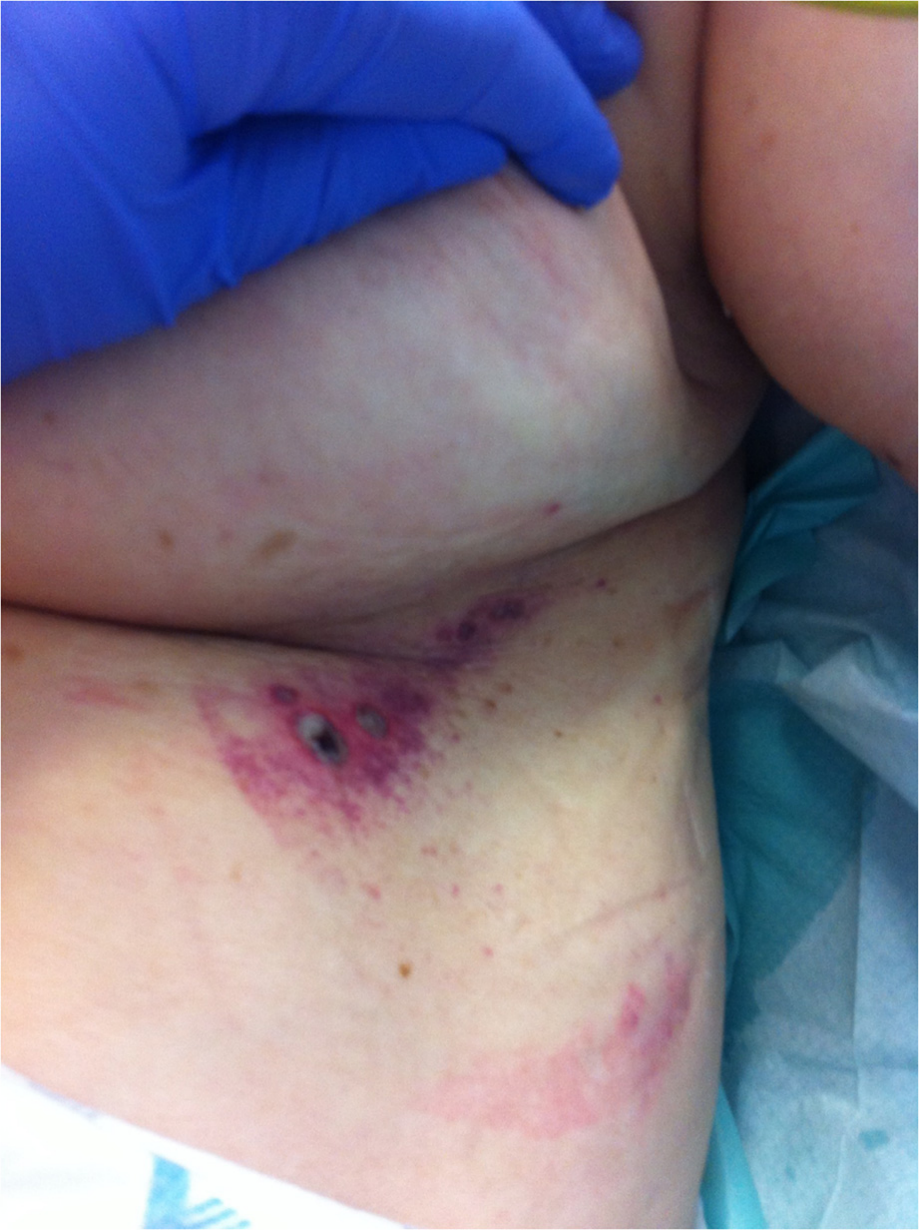
**Two little third-degree skin burns under the breast following use of an external cardiac pacing device.**

Electric cardioversion, defibrillation or ECPDs saves lives [1]. In particular, external pacing is indicated for untolerated bradyarrhythmia where medications have no effect. Classic complications of transcutaneous cardiac stimulation are related to electricity [2]. Indeed, electricity can lead to accidental injury of the medical team via lack of precautions and iatrogenic skin burns. However, no ECPD-related skin burn has been described for adults in the literature [3,4].

Although our patient benefited from the use of the ECPD, she acquired severe skin burns as a result of the treatment. Several possible explanations might explain this unusual complication. First of all, we cannot rule out that this issue resulted from a technical problem with the patch or glue. However, a poorly bonded patch or trapped air pockets between the gel and the skin could also be to blame. Additionally, greater impedance due to the breast weight of the patient could constitute a plausible explanation. Finally, the long wait time (2 hours) prior to placement of the internal pacing device might be a contributing factor.

Pain secondary to transcutaneous cardiac stimulation is usually due to electrically induced muscular contraction. Even though sedation-analgesia is indicated to prevent this common side effect, it can mask other complications. Skin burns must always be considered when patients continue to display pain under sedation-analgesia, especially in cases where the ECPD is used for a prolonged period of time.

## Abbreviation

ECPD: External cardiac pacing device
